# Recovery and Its Dynamics of Filamentous Fungi from Clinical Specimen Cultures: An Extensive Study

**DOI:** 10.1128/spectrum.00080-21

**Published:** 2021-08-04

**Authors:** Jian R. Bao, Ronald N. Master, Robert S. Jones, Richard B. Clark, Elizabeth C. Moore, Kileen L. Shier

**Affiliations:** a Quest Diagnostics Nichols Institute, Chantilly, Virginia, USA; Broad Institute

**Keywords:** filamentous fungi (molds), fungal culture, mold types, culture media, incubation time

## Abstract

The culture method remains vital in diagnosing fungal infections, but extensive data-based evaluation of the method, especially for filamentous fungi (molds), is minimal. The purpose of this study was to characterize mold recoveries from fungal cultures and the impact of media and incubation duration. Clinical specimens for fungal cultures were submitted primarily from the eastern and central United States, and mold isolation data were prospectively collected and analyzed. A total of 1,821 molds in 59 genera were isolated from 1,687 positive specimens, accounting for approximately 5.6% of our cohort of 30,000 fungal cultures. Within 2 weeks, nearly 90% of molds and 97.3% of Aspergillus fumigatus complex were recovered (>95% confidence interval [CI]). All *Mucorales* fungi were recovered within 11 days of incubation. The recovery peak time was day 3 for *Mucorales* fungi, day 4 for hyaline molds, day 5 for dematiaceous molds, and day 7 for *Onygenales* fungi. The recovery of Histoplasma capsulatum and *Trichophyton* species in the fourth week of incubation reveals that a 3-week incubation time is insufficient. Inhibitory mold agar was the best medium for recovering all mold types among all tested specimen types, yielding nearly 78% of mold growth overall, indicating the necessity of selective medium for fungal cultures.

**IMPORTANCE** Fungal culture is the gold standard method of diagnosing fungal infections, but important information, such as the impact of media and incubation times on fungal recovery, is not well documented. This study addressed these gaps using extensive data-based evaluation focused on molds. We identified the best medium types and incubation times for better fungal culture practice. We analyzed 1,821 molds from 1,687 positive specimens in our cohort of approximately 30,000 fungal cultures. Mold recovery peaked between 3 and 7 days of incubation, dependent upon the type of mold. Some well-defined fungal pathogens, such as Histoplasma capsulatum and *Trichophyton* species, were isolated in the fourth week of incubation. Inhibitory mold agar was identified as the best medium for recovering all mold types among all tested specimen sources. As we are aware, this is the largest study of fungal culture methods and supports 4 weeks of incubation for optimal mold recovery.

## INTRODUCTION

Culture remains the “gold standard” for laboratory diagnosis of fungal diseases, even as more culture-independent assays become available (1). Fungal infections are an increasing threat to human health. This is partially due to changing medical practices, such as the use of broad-spectrum antimicrobials and steroid therapies, an increase in immunocompromised patients, and an expanding list of fungal pathogens (2–4). Diagnostic challenges for detecting pathogenic fungi in clinical specimens remain, especially for those caused by filamentous fungi (5). The rapidly emerging worldwide spread of antifungal resistance requires obtaining viable fungal organisms for susceptibility testing, which is critical to patient care (3). Today, conventional culture still plays a vital role in diagnosing fungal infections and is important for patient management.

Optimal fungal culture protocols require data regarding medium selection and incubation duration, but these data are virtually lacking. Many fungal culture protocols are not data driven and may lead to inadequate pathogen detection (6). A deficiency of these data led to questions regarding the necessity of a 4-week incubation (6–8). Fungi recovered from the fourth week of incubation were considered clinically insignificant, and 3-week fungal culture had been suggested or implemented, including for cystic fibrosis patient specimens (6–9). A recent survey related to fungal cultures from cystic fibrosis specimens also showed that most clinical laboratories do not routinely use selective fungal culture media but, instead, use routine bacterial culture media for fungal cultures (10). With different protocols for fungal cultures in clinical laboratories, data to support these protocols are still limited, especially for recovery of filamentous fungal pathogens.

The objective of this study was to characterize the recovery of filamentous fungi based on the recovery times, numbers, types, and media on large specimen numbers, wide geographic coverage, and a year-long testing period and to provide fundamental information for constructing fungal culture protocols.

## RESULTS AND DISCUSSION

**FIG 1:**
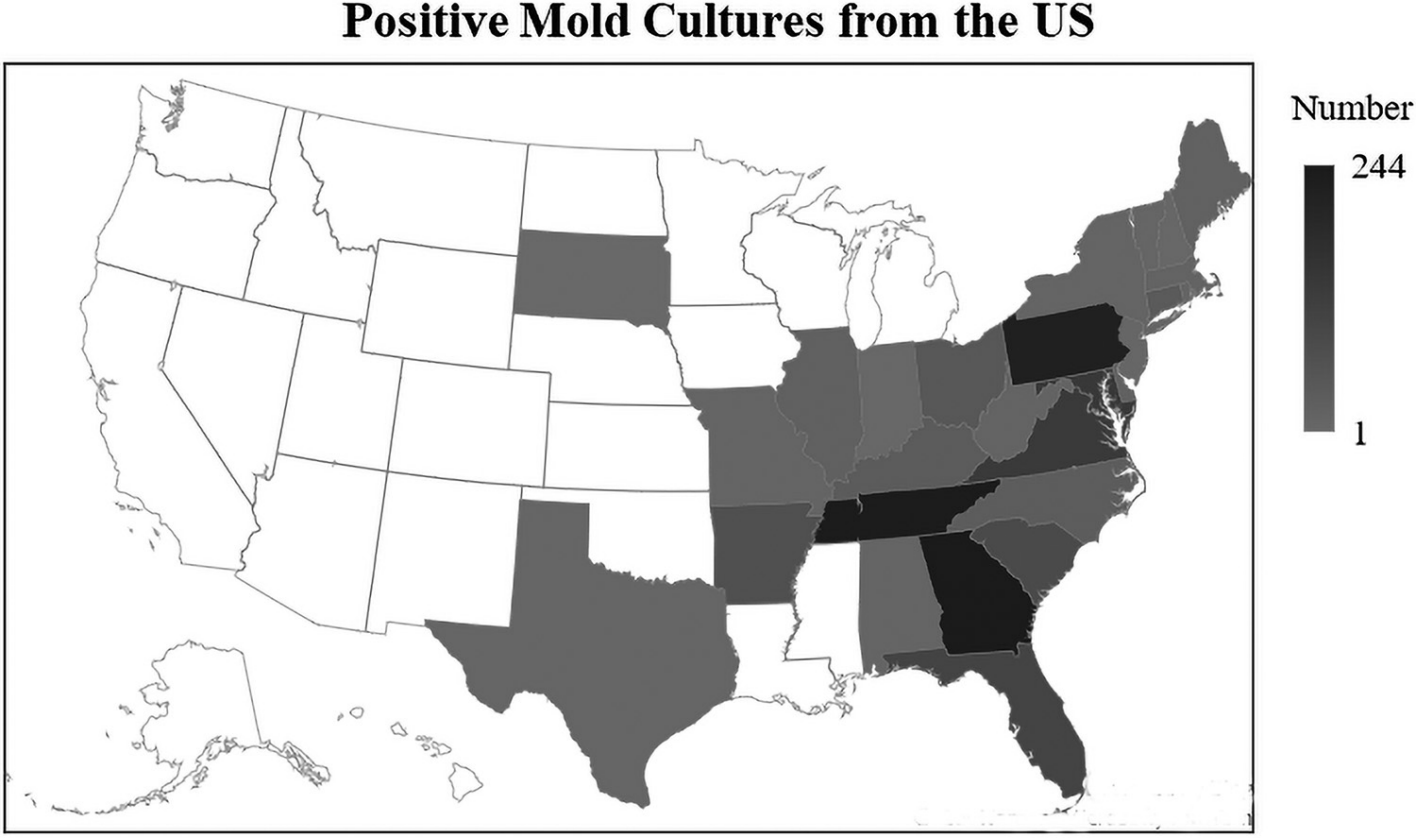
Geographic origins of the positive filamentous fungus culture specimens. Puerto Rico (18 positives) not shown.

More than 30,000 fungal cultures were examined in this study, and 1,687 specimens (approximately 5.6% of the total) were positive for filamentous fungi; 1,821 molds from 59 fungal genera and 7 *Actinomycetes* bacteria (*Nocardia* and *Streptomyces*) were recovered (Table 1 and see Table S1 in the supplemental material). The positive specimens were from 27 eastern and central continental states, the District of Columbia, and Puerto Rico (Fig. 1). Four molds were not isolated owing to heavy bacterial overgrowth. Among the positive cultures, 174 (10.3% of the total positive specimens) had two molds and 10 (0.6% of the total) had three molds isolated.

**TABLE 1:** Filamentous fungi recovered from culture specimens

Mold type	No. of isolates	%	No. of genera
Hyaline	1,251	68.7	21
A. fumigatus complex	185	14.8	
Other Aspergillus spp.	192	15.3	
Dematiaceous	308	16.9	31
*Onygenales*	229	12.6	3
*Mucorales*	29	1.6	4
Unable to identify	4	0.2	NA^*a*^
Total	1,821	100	59

a NA, not available.

The molds that produced structures were identified to the genus or species level by their morphological characteristics during the first week of subcultures, and approximately 25%, mostly dematiaceous molds, needed extended incubation to the next week for identification. Aspergillus was the most frequently recovered genus, representing approximately 30% of the hyaline molds (21% of the total) (Table 1). *Trichophyton* spp. were the predominant type (95.3%) among dermatophytes (*Onygenales* fungi).

Inhibitory mold agar (IMA) yielded the greatest recovery regardless of mold type and clinical specimen. Overall, 77.9% of molds were isolated from either IMA alone or in combination with Sabouraud dextrose agar Emmons (SDAE) or mycobiotic agar (MYA) (Table 2). IMA medium was superior in recovering dematiaceous molds compared to isolation of hyaline molds (70% versus 54.8%, respectively; >95% confidence interval [CI]). IMA medium alone accounted for 70.4% of molds recovered from skin source specimens and 50.1% of molds recovered from nonskin source specimens (Table 3). Molds recovered only from SDAE or MYA accounted for 22% of isolates.

**TABLE 2:** Mold recovery by source and medium types^*a*^

Specimen source	No. (%) of isolates^*b*^					
	IMA	SDAE	MYA	IMA and SDAE	IMA and MYA	Total
Nonskin	665	340	NC	322	NC	1,327
Respiratory	480	263	NC	212	NC	955
Tissues	27	11	NC	21	NC	59
Body fluids	12	10	NC	5	NC	27
Unspecified	146	56	NC	84	NC	286
Skin	345	1	57	3	84	490
Toe/nails	239	1	35	3	50	318
Hair	6	NC	2	NC	8	16
Unspecified	100	NC	20	NC	26	146
Total	1,010 (55.6)	341 (18.8)	57 (3.1)	325 (17.9)	84 (4.6)	1,817

a Actinomycetes (7 isolates) and nonrecoverable molds (4 isolates) are not included.

b IMA, inhibitory mold agar; SDAE, Sabouraud dextrose agar Emmons; MYA, mycobiotic agar; NC, no culture setup.

**TABLE 3:** Mold types recovered on medium^*a*^

Medium^*b*^	No. (%) of isolates				
	Hyaline	Dematiaceous	*Onygenales*	*Mucorales*	Total
IMA only	642	216	115	20	993 (54.8)
SDAE only	294	39	8	2	343 (19.0)
MYA only	13	4	40	1	58 (3.2)
IMA and SDAE	278	42	6	6	332 (18.3)
IMA and MYA	16	4	65	0	85 (4.7)
Total	1,243 (68.7)	305 (16.8)	234 (12.9)	29 (1.6)	1,811

a Actinomycetes (7) and nonrecoverable molds (4) are not included.

b IMA, inhibitory mold agar; SDAE, Sabouraud dextrose agar Emmons; MYA, mycobiotic agar.

The superior performance of IMA in mold isolations compared to that of the other tested media supports the previous results from a smaller study (11, 12). IMA is an enriched selective fungal medium containing a broad-spectrum antibiotic, chloramphenicol. Its excellent performance in mold isolation is likely associated with the antibiotic contribution to its effective inhibition of bacterial growth. SDAE, a nonselective nutrient medium that is generally considered the primary medium for fungal isolation from clinical specimens, did not perform as well as expected. We observed bacterial overgrowth on this medium more often than on the selective media, which could be the factor that deterred mold growth. Despite the primary indication of MYA for the recovery of dermatophytes, IMA performed better than MYA in isolating *Onygenales* fungi (Table 3). Though cost containment in clinical laboratories has become an unavoidable issue, our data demonstrate the need to include a selective medium for fungal culture or to send specimens to a clinical laboratory equipped with a selective medium for better fungal isolation. The addition of a selective medium to fungal culture may be especially important for specimens from cystic fibrosis patients (7).

Thermally dimorphic molds cause more than one million new infections each year in the United States alone (13). Ten dimorphic fungal isolates were recovered in this study from lymph nodes, body fluids, tissues, and respiratory specimens. They included five Histoplasma capsulatum isolates, two Blastomyces dermatitidis isolates, two Sporothrix schenckii isolates, and one *Coccidioides* sp. Nine were recovered from IMA alone and one (Coccidioides immitis*/*posadasii) was obtained on SDAE alone. Their average recovery time was 13 days; the shortest was a *Histoplasma* isolate recovered at day 5, and the longest was also a *Histoplasma* sp. isolated at day 30.

*Mucorales* fungi are important human pathogens owing to their rapid, progressive, and destructive nature (14, 15) and are the most rapidly growing groups of filamentous fungi. Their recovery peaked at day 3, and 88% of 29 *Mucorales* molds were isolated during the first incubation week in this study. No *Mucorales* molds were recovered after 11 days. These data suggest that the cultures to recover *Mucorales* molds can be completed within 2 weeks. The short recovery window for these molds also suggested that the *Mucorales* molds might be fastidious and labile in *in vitro* specimens and supported the guidelines that prompt culturing of these fungal specimens is vital for their recovery (14).

**FIG 2:**
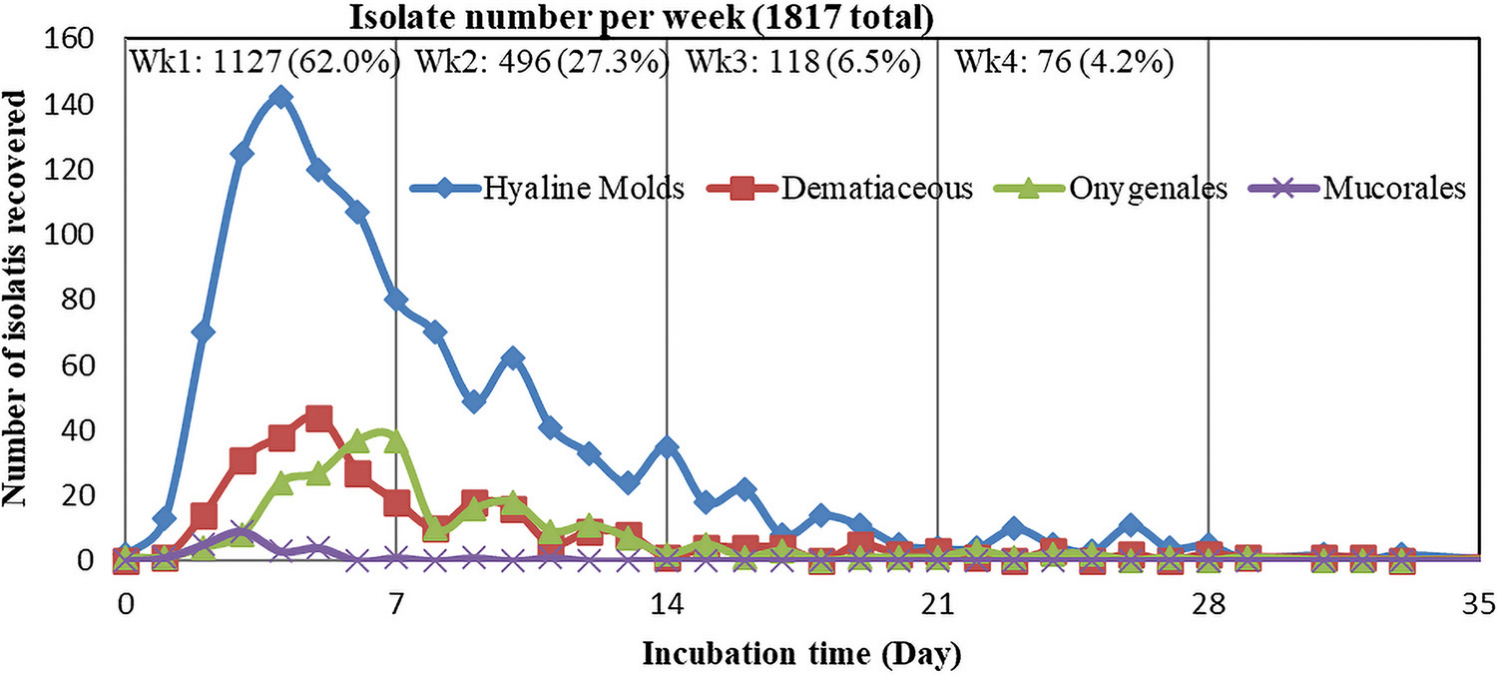
Number of molds recovered over incubation time.

Most molds were recovered in 1 (62%) or 2 weeks (89%) (Table 4) (>95% CI), and their different recovery peak times largely reflected their different growth rates on media. Mold recovery peaked on day 3 for *Mucorales* fungi, day 4 for hyaline molds, day 5 for dematiaceous molds, and day 7 for *Onygenales* fungi (Fig. 2). More than 95% of Aspergillus spp., the most frequently isolated mold type, were recovered in the first 2 weeks of incubation. Aspergillus fumigatus complex is typically rapid growing and was recovered at 85.4% in the first week and at 97.3% within the first 2 weeks (>95% CI). The time to recovery was independent of the culture media used.

**TABLE 4:** Molds recovered from week 4 incubation

Mold type (*n*)	No. of isolates	Main source
Hyaline molds (50)		
Aspergillus fumigatus complex	2	BW^*a*^ and other respiratory
Other Aspergillus spp.	4	Various
* Acremonium* sp.	1	Nail
* Paecilomyces* spp.	3	Respiratory
* Penicillium* spp.	14	BW or others
* Sporotrichum* sp.	1	BW
Sterile hyphae	25	BW or others
Dematiaceous (12)		
Dematiaceous molds	5	BW or others
* Doratomyces* spp.	4	BW or skin
* Curvularia* sp.	1	Nail
* Cladosporium* sp.	1	BW
* Epicoccum* sp.	1	BW
Dimorphic (3)		
Histoplasma capsulatum	3	Lymph node, BW, or unknown
*Onygenales* (10)		
* Trichophyton* spp.	10	Toe/nail, skin, scalp
*Mucorales* (0)	0	
Total	76	

a BW, bronchial washing.

Three Histoplasma capsulatum and 10 *Trichophyton* isolates were recovered during the fourth week of incubation. One Histoplasma capsulatum isolate was recovered on day 30, and the other two were recovered on day 22, which could be assigned into week-3 isolations based on our culture reading scheme. These organisms are likely clinically significant as they are well-recognized human pathogens, though their relations to clinical scenarios were unknow in this study owing to lack of access to the clinical data. In this study, 76 isolates were recovered from the cultures in the fourth week of incubation, or 4.2% of the total isolate number, but 13 of them (approximately 17% from the fourth week) were probably clinically relevant (Table 4). Despite a prior study suggesting adequate recovery of molds within 3 weeks (8), our study recovered frank fungal pathogens during the fourth week of incubation, indicating the inadequacy of 3-week incubation for fungal culture. A 4-week incubation period should improve mold detection, reducing the need for duplicate fungal orders. In addition, the data provided in this study can be used to design specific fungal culture algorithms based on specimen types and to target specific fungal organism recoveries (14).

One of the limitations in this study was the lack of access to patients’ clinical data. Another shortcoming is that most of the isolates originated from the eastern United States, limiting the recovery of fungal pathogens more common in the western United States., e.g., *Coccidioides* (Fig. 1). Yeast isolation data were not included in this study by design. The study provides valuable results with large data elements to determine optimal fungal culture procedures, such as the incubation duration, medium selection, and differential protocol designs as mentioned above. This will help to improve fungal culture performance, enhance decision making in clinical laboratories, adopt appropriate fungal culture practices, and thus translate diagnostic data into better patient care.

## MATERIALS AND METHODS

Filamentous fungal recovery data were prospectively collected between 2018 and 2019. The fungal cultures were performed from clinical specimens that were submitted from clinician offices, hospitals, or other clinical laboratories of 27 locations in the eastern and central United States, Washington, DC, and Puerto Rico. Specimens were processed and cultured according to standard laboratory protocol guidelines (16), using aseptic technique to minimize environmental contaminants. Fungal blood cultures were not included in this study.

Three culture media, on slants, were used for fungal culture: Sabouraud dextrose agar Emmons (SDAE; Becton Dickson [BD], NJ), inhibitory mold agar (IMA; BD), and mycobiotic agar (MYA; Thermo Fisher Scientific, San Diego, CA). SDAE is a nonselective fungal medium, while IMA and MYA are selective fungal media, with the former supplemented with the antibiotic chloramphenicol and the latter supplemented with both chloramphenicol and cycloheximide to suppress bacterial and fungal growth. Each specimen was cultured on two of the three media, depending on the specimen source. Nonskin source specimens (i.e., respiratory, body fluids, and tissues) were inoculated onto both IMA and SDAE. Skin, hair, and nail specimens were inoculated onto both IMA and MYA. The cultures were incubated at 30°C and examined twice weekly for the first 2 weeks and then once a week for an additional 2 weeks. The mold recovery time was measured from the day specimens were processed and inoculated onto the media to the day when mold growth was first observed.

Colonies were subcultured onto potato flake agar (BD, Baltimore, MD) and incubated at 30°C. Both colony and microscopic characteristics were examined for morphological identification (11). The identification, when necessary, was aided by using biological characteristics or methods, such as growth temperature or chemical utilizations, or by fungal internal transcribed spacer (ITS) region DNA sequencing. Morphological identifications for dimorphic molds were confirmed using the DNA sequencing method. Molds that did not produce identifiable structures such as spores or fruiting bodies within 2 weeks of subculturing were recorded as either dematiaceous fungi (for pigmented molds) or sterile hyphae (for hyaline molds). As recommended by the Clinical and Laboratory Standards Institute (17), we used more commonly and clinically recognized names for molds in this study.
